# Life-threatening intoxication with methylene bis(thiocyanate): clinical picture and pitfalls. A case report

**DOI:** 10.1186/1471-227X-6-5

**Published:** 2006-04-11

**Authors:** Claude Braun, Rainer Birck, Manfred V Singer, Peter Schnuelle, Fokko J van der Woude, Matthias Löhr

**Affiliations:** 1 Department of Medicine V (Nephrology/Endocrinology/Rheumatology), University Medical Center Mannheim, University of Heidelberg, D-68135 Mannheim, Germany; 2 Department of Medicine II (Gastroenterology/Hepatology/Infectious Diseases), University Medical Center Mannheim, University of Heidelberg, D-68135 Mannheim, Germany; 3 Départment de Médecine Interne et Néphrologie, Centre Hospitalier Kirchberg, L-2540 Luxembourg

## Abstract

**Background:**

Methylene bis(thiocyanate) (MBT) is a microbiocidal agent mainly used in industrial water cooling systems and paper mills as an inhibitor of algae, fungi, and bacteria.

**Case presentation:**

We describe the first case of severe intoxication following inhalation of powder in an industrial worker. Profound cyanosis and respiratory failure caused by severe methemoglobinemia developed within several minutes. Despite immediate admission to the intensive care unit, where mechanical ventilation and hemodialysis for toxin elimination were initiated, multi-organ failure involving liver, kidneys, and lungs developed. While liver failure was leading, the patient was successfully treated with the MARS (molecular adsorbent recirculating system) procedure.

**Conclusion:**

Intoxication with MBT is a potentially life-threatening intoxication causing severe methemoglobinemia and multi-organ failure. Extracorporeal liver albumin dialysis (MARS) appears to be an effective treatment to allow recovery of hepatic function.

## Background

Extracorporal albumin dialysis using the Molecular Adsorbent Recirculating System (MARS^®^) is now used in many hospitals to support excretory hepatic function. Main indications are acute-on-chronic liver failure, acute liver failure, primary graft dysfunction after liver transplantation, and liver failure post liver surgery [1-3]. The MARS procedure allows removal of the protein-bound toxins, such as bilirubin, phenols, or "false" neurotransmitters, improvement of hepatorenal syndrome, and the creation of a "bridge" for patients awaiting urgent liver transplantation. Theoretically this treatment is suggestive to be of value in intoxications in which the liver represents the main target organ, either by eliminating protein-bound substances not removed by other extracorporeal procedures, or by supporting excretory hepatic function, thereby allowing spontaneous recovery of the failing liver or transplantation of a suitable graft. Here we report an unusual case of acute liver failure secondary to intoxication with MBT successfully treated with MARS.

## Case presentation

A 39 year old industrial worker with no former medical history suddenly complained of nausea, dizziness, respiratory tract irritation, and dyspnea during sweeping several kilograms of a powder that had escaped from a barrel containing methylene bis(thiocyanate) (MBT). The worker was not wearing any personal protective equipment. Profound cyanosis developed, and the patient was admitted in our hospital, where mechanical ventilation with 100% oxygen was initiated. 4-dimethylaminophenol (4-DMAP; 250 mg) followed by sodium-thiosulfate (6000 mg) were immediately administered intravenously for treatment of assumed cyanide intoxication. Arterial blood gas examination by co-oxymeter performed a few minutes later revealed the following arterial-blood gas values: pH 7.39, partial pressure of carbon dioxide 37 mmHg, partial pressure of oxygen 381 mmHg, oxygen saturation 63%, methemoglobin level of 51%, oxyhemoglobin level of 48%, carboxyhemoglobin level 0.4%. The i.v. administration of tolonium chloride (toluidine blue, 300 mg, repeated once within 30 minutes) lowered the methemoglobin level to 12% within one hour. The patient was transferred to the medical ICU. In the meantime information on MBT toxicity was requested from several German Intoxication centers. Given the lack of a specific antidot, the experts at these centers recommended treatment with hemodialysis for toxin elimination in addition to general supportive care and monitoring of renal, hepatic and pulmonary function. Only very limited data, however, provided the rationale for these measures, and most information was obtained from anecdotal reports of intoxication with methyl-isocyanate, a chemical analogue of MBT (the accidental release of a methyl-isocyanate cloud (composed of phosgene and isocyanate) was implicated in the Bhopal, India, disaster in 1984) [4,5]. Laboratory and pulsoxymetric evaluation were markedly impeded by the bluish discoloration of skin and serum samples after toluidine blue administration. Daily hemodialysis treatment was started. On the second day a slight increase in renal retention parameters and transaminases was observed, together with massive hemolysis (hemoglobin decrease from 14.5 to 8.8 g/dl, LDH > 6000 U/l, haptoglobin < 0.3 g/l) and an increase of methemoglobin from 13 to 34%, all attributed to MBT toxicity. A total of seven units of packed red cells were administered. In the following days, anuric acute renal failure and adult respiratory distress syndrome developed, making the initiation of continuous veno-venous hemofiltration and adoption of a more aggressive ventilation strategy necessary. Hemolysis recovered spontaneously, but the further course of intensive care treatment was complicated by progressive hepatic failure with prominent hyperbilirubinemia and increasing liver function impairment (TBIL 16.5 mg/dl, DBIL 13.0 mg/dl, ALAT 50 U/l, ASAT 49 U/l, CHE 2820 U/l, prothrombine time 51%, INR 1.83, aPTT 34.9 sec, fibrinogen 2.03 g/l, AT III 81%). Extrahepatic cholestasis was excluded by ultrasonographic examination, and no clinical or laboratory signs of sepsis could be found. Three sessions of treatment with MARS (MARS monitor, Teraklin AG, Rostock, Germany) were performed on days 17, 18, and 22 after hospital admission in combination with ordinary hemodialysis (F8 HPS hemofilter, dialysate flow 800 ml/min, Fresenius 4008H, Fresenius Medical Care AG, Bad Homburg, Germany) using albumin dialysate and blood flow rates of approximately 150–180 ml/min for 7 to 8 hours each session. Anticoagulation with unfractionated heparin was monitored by activated clotting time (target range: 160–180 seconds). After the first procedure the bilirubin level decreased by 46%. The final bilirubin level after the third MARS session was 6.0 mg/dl. Liver function and the general condition then recovered, and the patient could be extubated 2 days after the third liver dialysis treatment. Due to persisting anuric renal failure, a permanent dialysis catheter for chronic hemodialysis treatment was implanted. The patient was discharged on a peripheral ward on day 36 after hospital admission. Renal function slowly recovered over the next days, and the patient could finally be withdrawn from hemodialysis program on day 44 of his hospital stay. The further course was complicated by a severe peripheral neuropathy that was characterised electrophysiologically by a predominantly axonal damage pattern. Differential diagnosis included critical-illness neuropathy and MBT-mediated neurotoxicity. The patient was transferred to a rehabilitation centre. Final blood chemistry at our hospital included following results: creatinine 1.42 mg/dl, urea 62 mg/dl, TBIL 2.7 mg/dl, ALAT 125 U/l, ASAT 59 U/l, CHE 3667 U/l, hemoglobine 9.1 g/dl, LDH 175 U/l, and prothrombine time 99 % (Table 1).

**Table 1:** Selected Blood Chemistry Before and After Treatment with MARS Albumin Liver Dialysis

	At admission (day 0)	Before first MARS session (day 16)	After first MARS session (day 17)	After last MARS session (day 23)	At discharge (day 69)
TBIL (mg/dl)	0.6	16.5	8.9	5.9	2.7
DBIL (mg/dl)	n.a.	13.0	n.a.	3.1	n.a.
ALAT (U/l)	23	50	n.a.	79	125
ASAT (U/l)	17	49	n.a.	34	59
Prothrombine time (%); [INR]	90 [0.98]	68 [1.51]*	64 [1.56]	95 [0.97]	99 [0.96]
CHE (U/l)	5577	2820	2237	2766	3667
Creatinine (mg/dl)	1.04	5.70	2.78	3.48	1.42

* 4 units of fresh frozen plasma had previously been administered.

TBIL: total bilirubine; DBIL: direct bilirubine; ALAT: alanine aminotransferase; ASAT: aspartate aminotransferase; INR: international normalized ratio; CHE: cholinesterase; n.a.: not available

## Conclusion

Methylene bis(thiocyanate) (MBT) is used as a biocide in a number of industrial and residential applications [6,7]. A chemical overview is presented in Table 2. MBT was first registered in the US in 1949 as an active ingredient. Currently 49 active products are registered in the Office of Pesticide Programs Database [7,8], such as adhesives, coatings, fuels and various other speciality industrial products primarily used as a preservative and as an inhibitor of algae, fungi, and bacteria in reverse osmosis and cooling water systems, leather processing and paper mills. It is also used as a protection treatment of wood and wood surfaces and is not limited in its efficacy by the presence of organic matter and oils. MBT consists of a methane group containing two thiocyanate groups, presenting as a yellow granular solid with sulphur like smell. Toxicological database is far from being complete for all current use patterns [6,7,9]. A hazard has been identified for inhalation exposure, since harmful concentrations of airborne particles can be reached quickly. Furthermore MBT can cause severe skin irritation. Persons handling this product are therefore advised to wear goggles or face shields and rubber gloves. Dispersion of MBT containing dust must be avoided.

**Table 2:** Chemical Overview of Methylene (Bis)Thiocyanate

Common name:	Methylene bis(thiocyanate)
	Methylene dithiocyanate
	Thiocyanic acid, methylene ester
Chemical name:	Methylene bis(thiocyanate)
CAS Registry Number:	6317-18-6
OPP Chemical Code:	068102
Chemical formula:	C_3_H_2_N_2_S_2_
Structural formula:	SCN-CH_2_-SCN
Molecular weight:	130.19
Trade and other names (USA):	Slimicide MC
	Busan 110
	Nalco D-1994
	Cytox
Basic manufacturers (USA):	Akzo Chemicals, Inc.
	Albright and Wilson, Ltd.
	Buckman Laboratories International, Inc.

There is still a data gap for metabolism primarily due to inadequate metabolite idenfication [7]. In rats, up to 99% of [^14^C]MBT were excreted over a 4-day period. Urine was the primary route of excretion (55–70%), followed by fecal elimination (15–30%) and expired CO_2 _(10–15%). It is tempting to speculate that fecal elimination is most likely achieved via biliary excretion of MBT and/or toxic metabolites. This would help to explain the cholestatic pattern of acute liver failure observed in our patient. Radiolabeled compounds found in fecal extracts from rats treated with high, but not in those with low, doses were identified as [^14^C]MBT (Brown L et al., Inveresk Research International, unpublished data). However the data provided on the identification of the metabolites in the urine, feces, expired air, and tissue is incomplete. Total radioactivity in the blood, blood cyanide and plasma thiocyanate exhibited biphasic elimination from the blood. The terminal elimination phase started 1 hour after dosing and had a half-life of 7 hours. The following mechanisms of metabolism were proposed: One is the reaction of alkylmono-thiocyanates with a soluble liver enzyme fraction containing glutathione S-transferase and glutathione. A second reaction involves P-450 isoenzymes. The additional thiocyanate group may make the methylene carbon suitable for oxygen insertion producing the corresponding aldehyde with the release of thiocyanate. Further oxidation of the aldehyde to the corresponding acid followed by decarboxylation would yield CO_2_. Toxicity of MBT would therefore be due to 1) cyanide release, 2) glutathione depletion, and 3) toxic metabolites other than cyanides and thiocarbamates. Hydrolysis studies indicate that MBT degrades to thiocyanate ion, formic acid, and mercapto-(methylenethiocyanate) depending on the pH of the medium. So far, however, it is not clear whether degradation to cyanide occurs in the working environment. In the present case the latter possibility could be excluded by biochemical analysis of a serum specimen obtained at the time of hospital admission, in which no cyanide could be detected. In addition patients with acute cyanide intoxication typically develop severe lactic acidosis, which was not observed in the present case.

Toxicity studies in rats and mice reported signs and symptoms comparable to those of acute cyanide intoxication and included dyspnea, tremors, and ataxia after MBT doses up to 160 mg/kg body weight [10]. In animals surviving for at least 24 hours, the stomach was identified as the target organ, demonstrating necrotic inflammatory lesions of the mucosal surface. After 13 week administration rats treated with MBT (up to 16 mg/kg) developed mild anemia, and sperm motility was decreased in male rats. No mutagenic properties were observed in S. typhimurium, with or without S9 activation.

Two occupational incidents were reported to the Office of Pesticide Programs Incident Data System [7]. The first involved a man who was exposed to MBT while dipping lumber. He developed swollen eyes, a rash on his cheeks and insomnia. The man was diagnosed lupus erythematodes during follow-up; a causal relationship to the pesticide was considered improbable. In the second incident, a man was exposed to a multiple active ingredient formulation containing 10% MBT through a leaking line. He developed a rash on his arm, shortness of breath and a cough, but fully recovered within 24 hours. Clearly the patient reported here had a much more severe course after inhalational exposure to MBT, exhibiting a biphasic pattern with methemoglobinemia and hemolytic anemia immediately after hospital admission and life-threatening multi-organ failure involving liver, kidneys and lungs in the following days. Although we cannot formally prove a causal relationship on the basis of the present case description, inhalational MBT uptake most likely accounts for the described complications. Glucose-6-phosphate-dehydrogenase deficiency, known to exaggerate methylene blue toxicity and cause acute hemolysis, was excluded later during the hospital stay. The molecular basis of MBT toxicity remains unclear, although it is most probably not related to the production of cyanide, but rather to methemoglobinemia.

This case is, to our best knowledge, the first report of a multi-organ failure secondary to inhalation of methylene-bis-thiocyanate and one of the first reports of the successful use of MARS albumin dialysis in hepatic failure due to intoxication [11-13]. This case underlines the diagnostic and therapeutic dilemma of emergency physicians faced with unusual intoxications. A direct effect of MARS on MBT metabolism or on toxin elimination is very unlikely, as our patient has been treated with MARS only after more than 14 days of extracorporeal renal replacement therapy. MARS albumin dialysis therefore appears to be an effective treatment to allow recovery of liver function after severe intoxication or, in the case of more fulminant liver disease, bridge patients awaiting urgent liver transplantation.

## Declaration of competing interests

The author(s) declare that they have no competing interests.

## Authors' contributions

ML, MVS and PS were responsible for the treatment of the described patient including MARS liver albumin dialysis. FJW and CB performed the extensive literature and database research on MBT and its toxicity. CB and RB drafted the manuscript, which was read and approved by all authors in its final version.

## Pre-publication history

The pre-publication history for this paper can be accessed here:


